# Anticholinergic burden and bone health in rheumatoid arthritis

**DOI:** 10.1590/1806-9282.20260191

**Published:** 2026-07-24

**Authors:** Bilal Uysal, Ender Salbaş, Nilay Şahin

**Affiliations:** 1 Balıkesir University, Faculty of Medicine, Department of Physical Medicine and Rehabilitation – Balıkesir, Türkiye.

**Keywords:** Arthritis, rheumatoid, Osteoporosis, Polypharmacy, Cholinergic antagonists, Risk assessment

## Abstract

**OBJECTIVE:**

Rheumatoid arthritis is associated with osteoporosis and falls. While anticholinergic burden increases fracture risk in older adults, its contribution to bone health in rheumatoid arthritis is unclear. The aim of this study was to determine if anticholinergic burden is an independent risk factor for osteoporosis and fracture in rheumatoid arthritis.

**METHODS:**

This retrospective cohort study included 120 adults with rheumatoid arthritis. Cumulative anticholinergic burden was quantified using eight validated scales (anticholinergic burden, Anticholinergic Risk Scale, Anticholinergic Drug Scale, Clinician-rated Anticholinergic Scale, Anticholinergic Activity Scale, Anticholinergic Effect on Cognition Scale, Anticholinergic Loading Scale, and CRIDECO). Outcomes included lumbar spine and hip bone mineral density, Fracture Risk Assessment Tool 10-year fracture probabilities, and World Health Organization-defined osteoporosis. Fracture Risk Assessment Tool major and L1-L4 T-score models included age, body mass index, rheumatoid arthritis duration, anticholinergic burden Calc Score, and Anticholinergic Drug Scale Score, whereas the binary osteoporosis model included age, body mass index, comorbidity count, anticholinergic burden Calc Score, and Anticholinergic Drug Scale Score.

**RESULTS:**

In unadjusted analyses, only Clinician-rated Anticholinergic Scale ≥1 was associated with osteoporosis (p=0.010), whereas the other thresholdbased scales were not. After adjustment for the covariates specified in each model, neither anticholinergic burden Calc Score nor Anticholinergic Drug Scale Score independently predicted Fracture Risk Assessment Tool major probability (p=0.415 and p=0.189), L1–L4 T-score (p=0.436 and p=0.291), or binary osteoporosis status (p=0.849 and p=0.653). Age remained the only independent predictor in the osteoporosis model (OR 1.083; 95%CI 1.019–1.151; p=0.010), whereas older age and lower body mass index predicted higher Fracture Risk Assessment Tool major probability.

**CONCLUSION:**

In rheumatoid arthritis, the apparent relationship between anticholinergic burden and osteoporosis is largely explained by age, body composition, and comorbidity-related prescribing. Although anticholinergic burden was not an independent fracture risk factor after adjustment, higher anticholinergic burden may still reflect greater prescribing complexity and comorbidity burden in rheumatoid arthritis.

## INTRODUCTION

Rheumatoid arthritis (RA) is an independent risk factor for osteoporosis and fragility fractures, with approximately twice the risk of the general population due to systemic inflammation and medication use^1,2,3^. Since patients with RA also have a high prevalence of falls (34%), they are uniquely susceptible to skeletal injury^4^. This vulnerability necessitates scrutiny of iatrogenic contributors. RA management often involves complex regimens for comorbidities like fibromyalgia and gastrointestinal disorders, leading to polypharmacy and cumulative anticholinergic burden (ACB)^5,6^. High ACB is linked to central nervous system adverse effects, unsteadiness, and falls^7,8,9,10,11^. While high ACB increases fracture risk by 19-49% in the general geriatric population^6,7,8^, its interaction specifically within RA remains underexplored. It is unclear whether ACB adds independent risk amidst existing disease vulnerability. This study examined whether ACB, measured by multiple validated scales, is independently associated with bone mineral density (BMD) and FRAX-based fracture risk in an RA cohort.

## METHODS

### Study design and participants

This retrospective cohort study analyzed 120 adult patients with RA meeting the 2010 ACR/European League Against Rheumatism classification criteria who were followed at a tertiary physical medicine clinic^12^. Inclusion required complete dual-energy X-ray absorptiometry (DXA) and Fracture Risk Assessment Tool (FRAX) data, with FRAX calculated, including femoral neck BMD^13^. Patients with incomplete data, malignancy, or secondary osteoporosis causes were excluded. The study was approved by the Institutional Ethics Committee, waiving informed consent due to the retrospective design.

The study protocol was approved by the Institutional Ethics Committee of Balıkesir University (BAUN 2024/209; 03 December 2024).

### Data collection and variables

Data were extracted from electronic health records, including:

Clinical: Age, sex, BMI, RA duration, serology (rheumatoid factor/anti-cyclic citrullinated peptide), and treatment duration.

Anticholinergic Burden Assessment: Quantified using eight validated scales: ACB^14^, ARS^15^, ADS^16^, CRAS^17^, AAS^18^, AEC^19^, ALS^20^, and CRIDECO^11^.

Treatment: Categorized into conventional synthetic (csDMARDs), biologic (bDMARDs), and targeted synthetic DMARDs (tsDMARDs).

Bone health: FRAX 10-year probability (major osteoporotic/hip fracture) and BMD T-scores (lumbar spine L1–L4 and femoral neck). Osteoporosis was defined by the World Health Organization (WHO) criteria (T-score≤-2.5).

Comorbidity count: Defined as the total number of documented major chronic conditions per patient, such as hypertension and gastrointestinal disorders.

For threshold-based analyses, ACB ≥2 was used to indicate at least moderate anticholinergic burden, whereas thresholds of ≥1 on the remaining scales indicated the presence of any anticholinergic exposure, in line with their original validation studies^14^.

### Statistical analysis

Analyses were performed using Python (v3.9). Normality was assessed via the Shapiro-Wilk test. Non-parametric tests (Kruskal-Wallis, Spearman’s correlation) were used for non-normally distributed variables. Associations between categorical ACB thresholds and osteoporosis were assessed using Pearson chisquare tests. Multivariable linear regression identified predictors of FRAX major probability and L1–L4 T-scores, and logistic regression evaluated predictors of osteoporosis (binary).

The FRAX major probability and L1–L4 T-score models included age, BMI, RA duration, ACB Calc Score, and ADS Score. The binary osteoporosis model included age, BMI, comorbidity count, ACB Calc Score, and ADS Score.

A post-hoc power analysis (using G*Power v3.1) confirmed >80% power to detect a medium effect size (f^2^=0.15) for multivariable regression with up to five predictors (α=0.05). For multivariable modeling, the Anticholinergic Cognitive Burden (ACB) and Anticholinergic Drug Scale (ADS) were selected a priori as representative scales because of their continuous nature and widespread clinical validation to avoid multicollinearity.

## RESULTS

### Patient characteristics

The cohort (n=120) was predominantly female (91.7%) with a mean age of 61.3±8.6 years. Most received csDMARDs (58.3%), followed by bDMARDs (35%) and tsDMARDs (6.7%). Patients on advanced therapies (bDMARDs/tsD-MARDs) had significantly longer disease duration (p=0.012) but similar age (p=0.368) compared to the csDMARD group (Table 1). Osteoporosis was present in 18.5% of patients.

**Table 1:** Baseline demographics and clinical characteristics by treatment group (n=120).

Characteristic	Overall (n=120)	csDMARD (n=70)	bDMARD (n=42)	tsDMARD (n=8)	p-value
Age, years (mean±SD)	61.3±8.6	60.6±8.7	62.9±8.9	59.9±4.2	0.368
Sex, female n (%)	110 (91.7%)	64 (91.4%)	38 (92.7%)	7 (87.5%)	0.835
BMI, kg/m^2^ (mean±SD)	29.7±5.4	29.0±5.6	30.2±4.7	33.4±5.0	0.053
RA duration, years (mean±SD)	9.1±5.6	7.8±5.1	10.9±5.9	12.0±5.2	0.012
Treatment duration, years (mean±SD)	8.6±5.5	7.4±5.2	10.5±6.0	10.8±6.0	0.017
Drug count (mean±SD)	5.6±2.3	5.8±2.3	5.6±2.3	5.0±1.9	0.512
Osteoporosis, n (%)	22 (18.5)	11 (15.7)	10 (24.4)	1 (12.5)	0.469
Hypertension, n (%)	74 (61.7)	42 (60.0)	28 (68.3)	4 (50.0)	0.528
GI issues, n (%)	52 (43.3)	30 (42.9)	20 (48.8)	2 (25.0)	0.635

Notes: Values are presented as mean±SD or n (%). p-values were derived from the Kruskal-Wallis test for continuous variables and the chi-square test for categorical variables. csDMARD: conventional synthetic DMARD; bDMARD: biologic DMARD; tsDMARD: targeted synthetic DMARD; BMI: body mass index; RA: rheumatoid arthritis; GI: gastrointestinal; SD: standard deviation.

### Anticholinergic burden and treatment

There was a significant inverse relationship between ACBs and advanced antirheumatic therapy. Spearman’s analysis showed all eight scales negatively correlated with treatment hierarchy; patients on bDMARDs and tsDMARDs had lower ACB. This was most pronounced for CRIDECO (r=-0.422, p<0.001) and ACB (r=-0.258, p=0.005) scores. Higher ACB also correlated with a higher total medication count but not specifically with RA medication intensity.

### Anticholinergic burden and bone health

In unadjusted analyses, most categorical ACB scores were not significantly associated with the diagnosis of osteoporosis (Table 2), with the exception of CRAS ≥1 (p=0.010). Continuous ACB scores were not significantly correlated with FRAX probabilities or BMD T-scores (all p>0.05).

**Table 2:** Unadjusted associations between anticholinergic burden and osteoporosis (World Health Organization categories).

Scale (score)	Threshold	n (≥threshold)	χ^2^	p-value
ACB (calc score)	≥2	30	0.720	0.698
ARS (drugs score)	≥1	14	5.113	0.078
ADS (score)	≥1	57	3.175	0.204
CRAS (score)	≥1	28	9.196	**0.010**
AAS (score)	≥1	11	1.232	0.540
AEC (score)	≥1	52	1.299	0.522
ALS (score)	≥1	52	1.299	0.522
CRIDECO (score)	≥1	95	0.363	0.834

Notes: Osteoporosis is defined by the World Health Organization categories (Normal/Osteopenia/Osteoporosis). Bold p-value indicates statistical significance (p<0.05). ACB: Anticholinergic Cognitive Burden; ARS: Anticholinergic Risk Scale; ADS: Anticholinergic Drug Scale; CRAS: Clinician-Rated Anticholinergic Score; AAS: Anticholinergic Activity Scale; AEC: Anticholinergic Effect on Cognition; ALS: Anticholinergic Load Scale; CRIDECO: CRIDECO Anticholinergic Load Scale.

### Independent predictors

After adjustment for the covariates specified in each model, neither ACB Calc Score nor ADS Score independently predicted FRAX major probability (p=0.415 and p=0.189), L1–L4 T-score (p=0.436 and p=0.291), or binary osteoporosis status (p=0.849 and p=0.653). Age remained the only independent predictor in the osteoporosis model (OR 1.083; 95%CI 1.019–1.151; p=0.010), whereas older age and lower BMI predicted higher FRAX major probability (Table 3).

**Table 3:** Multivariate linear and logistic regression models for predictors of bone health.

Model/predictor	β (coefficient)	Standard error	p-value	Odds ratio	95%CI
Dependent: FRAX major (R2=0.316, p<0.001)					
Age	0.312	0.047	**<0.001**	–	–
BMI	-0.179	0.076	**0.021**	–	–
RA duration	-0.045	0.072	0.534	–	–
ACB calc score	-0.391	0.477	0.415	–	–
ADS score	0.813	0.615	0.189	–	–
Dependent: L1–L4 T-score (R2=0.114, p=0.016)					
Age	-0.022	0.013	0.094	–	–
BMI	0.041	0.021	**0.050**	–	–
RA duration	-0.038	0.020	0.059	–	–
ACB calc score	0.103	0.131	0.436	–	–
ADS score	-0.180	0.169	0.291	–	–
Dependent: osteoporosis (binary) (pseudo R2=0.092, p=0.061)					
Age	0.080	0.031	**0.010**	1.083	1.019–1.151
BMI	-0.031	0.052	0.554	0.970	0.876–1.073
Comorbidity count	-0.236	0.195	0.227	0.790	0.538–1.158
ACB calc score	-0.061	0.318	0.849	0.941	0.505–1.754
ADS score	0.177	0.394	0.653	1.194	0.552–2.581

Note: β represents the unstandardized beta coefficient for linear regression models. Statistically significant predictors (p<0.05) are bolded. The binary osteoporosis logistic regression model should be interpreted cautiously because only 22 osteoporosis events were observed, limiting the eventsper-variable ratio. The FRAX model should also be interpreted as exploratory because FRAX already incorporates age, BMI, and rheumatoid arthritis in its calculation. FRAX: Fracture Risk Assessment Tool; BMI: body mass index; RA: rheumatoid arthritis; ACB: Anticholinergic Cognitive Burden; ADS: Anticholinergic Drug Scale; CI: confidence interval.

### Ancillary findings

Higher ACB was strongly linked to gastrointestinal comorbidities (χ^2^=15.975, p<0.001 for ACB scale ≥2).

## DISCUSSION

This study suggests that anticholinergic burden is not independently associated with skeletal fragility in ambulatory patients with RA. In unadjusted analyses, most threshold-based ACB measures were not associated with osteoporosis, and after multivariable adjustment, no independent association remained. In this context, ACB appears to function more as a marker of patient complexity and polypharmacy than as a direct skeletal risk factor^19^.

The higher ACB observed in patients on csDMARDs likely reflects a phenotype with greater symptom-driven prescribing for pain, gastrointestinal complaints, and related comorbidities rather than a direct effect of RA therapy itself^5,14^. Conversely, patients receiving bDMARDs had lower ACB, despite longer disease duration, which may reflect better inflammatory control and reduced need for adjunctive symptomatic medication. These findings differ from geriatric studies that linked ACB more directly to fracture outcomes^7,8^. In our ambulatory RA cohort, the influence of age and underlying disease burden may have overshadowed any pharmacologic contribution of ACB to skeletal outcomes^2,21^. Additionally, community-dwelling patients may experience fewer acute anticholinergic effects, such as delirium and falls, than more vulnerable institutionalized populations^22^.

Although ACB was not an independent fracture predictor in the adjusted models, higher ACB was associated with medication count and gastrointestinal comorbidity, supporting the interpretation that ACB may partly reflect prescribing complexity and comorbidity burden. In this context, ACB assessment may still support medication review, particularly in patients with polypharmacy or multiple comorbidities, without implying a direct skeletal effect^6,10^.

Several limitations merit emphasis. First, the retrospective single-center design limits causal inference and generalizability. Second, objective fall data were unavailable; because any effect of anticholinergic exposure on fracture risk is likely to be mediated largely through dizziness, sedation, and falls, the absence of fall history may have contributed to the null adjusted findings. Third, the binary osteoporosis model included only 22 events for five predictors, resulting in a low events-per-variable ratio and increased risk of overfitting; accordingly, the logistic regression findings should be interpreted cautiously. Fourth, the FRAX regression model should be viewed as exploratory rather than causal, because FRAX already incorporates age, BMI, and rheumatoid arthritis as an input variable set. Finally, disease activity measures (e.g., Disease Activity Score 28, C-reactive protein/erythrocyte sedimentation rate), cumulative glucocorticoid exposure, and concurrent antiresorptive or calcium/vitamin D therapy were not uniformly available for adjustment in this retrospective cohort and may have influenced the observed associations. Future prospective studies should incorporate fall history, frailty assessment, disease activity, glucocorticoid exposure, and treatment-stratified bone health outcomes^4^.

## CONCLUSION

In adults with RA, anticholinergic burden was associated with osteoporosis only in limited unadjusted comparisons and was not an independent predictor after accounting for relevant covariates. These findings suggest that ACB is better interpreted as a marker of comorbidity-related prescribing complexity than as a stand-alone fracture-risk factor. Even so, a high ACB may still identify patients who could benefit from medication review and broader fall-risk assessment.

## Data Availability

The datasets generated and/or analyzed during the current study are available from the corresponding author upon reasonable request.
